# Effect of N-arachidonoyl-(2-methyl-4-hydroxyphenyl) amine (VDM11), an anandamide transporter inhibitor, on capsaicin-induced cough in mice

**DOI:** 10.1186/1745-9974-2-2

**Published:** 2006-03-30

**Authors:** Junzo Kamei, Yuji Yoshikawa, Akiyoshi Saitoh

**Affiliations:** 1 Department of Pathophysiology & Therapeutics, School of Pharmacy and Pharmaceutical Sciences, Hoshi University, Tokyo 142–8501, Japan

## Abstract

**Background:**

Several observations have suggested that anandamide, an endogenous cannabinoid ligand, plays an important role in the modulation of cough sensitivity. However, it is unknown whether the anandamide membrane transporter plays a role in this modulation. To test this hypothesis, we investigated the effects of VDM11, an anandamide membrane transporter inhibitor, on capsaicin- and anandamide-induced cough.

**Methods:**

The effect of VDM11, an anandamide membrane transporter inhibitor, on capsaicin- and anandamide-induced cough in mice was examined.

**Results:**

VDM11, at doses of 3–10 mg/kg subcutaneously, produced a dose-dependent antitussive effect. This antitussive effect was antagonized by pretreatment with either intraperitoneal administration (3 mg/kg) or inhalation (1 mg/ml) of SR141716A, a cannabinoid receptor (CB1) antagonist. However, intracerebroventricular injection of SR141716A (0.03 mg/mouse) did not alter the effect of VDM11. Exposure of mice to a nebulized solution of 10% DMSO, a vehicle of anandamide, induced a cough response (7.7 ± 0.6 coughs/3 min; n = 10). Exposure of mice to a nebulized solution of anandamide, at concentrations of 0.03, 0.3 and 3 mg/ml, also produced a cough response in a concentration-dependent manner. The number of coughs induced by low dose (0.03 mg/ml) anandamide was significantly less than that of 10% DMSO. On the other hand, the number of coughs induced by high dose (3 mg/ml) anandamide was significantly greater than that of 10% DMSO. When AM251 (1.8 mM), a selective CB1 receptor antagonist, was given by aerosol for 4 min before inhalation of 0.03 mg/ml of anandamide, the number of coughs was significantly increased to the level observed with 10% DMSO alone. When capsazepine (0.3 mM), a selective TRPV1 receptor antagonist, was given via aerosol for 4 min before inhalation of 3 mg/ml of anandamide, the number of coughs was significantly decreased to the levels observed with 10% DMSO alone. The number of coughs induced by high dose (3 mg/ml) anandamide was significantly and dose-dependently reduced by the pretreatment with VDM11.

**Conclusion:**

These results suggest that anandamide, an endogenous cannabinoid ligand, may modulate cough sensitivity and that anandamide transporters play an important role in this modulation. Furthermore, these findings indicate that inhibition of the uptake of anandamide produced a potent antitussive effect and suggests that the anandamide transporter may be a potential target for peripherally acting antitussive drugs.

## Background

Anandamide, an endogenous cannabinoid ligand, is an ethanolamine amide of arachidonic acid that was first isolated from porcine brain and has been proposed to be an endogenous agonist of cannabinoid CB1 receptors [1]. On the other hand, several investigators have provided pharmacological and molecular evidence that anandamide is a full agonist at the vanilloid type 1 receptors (VR1, also known as transient receptor potential vanilloid 1 (TRPV1)), which are the sites of action of the pungent component of 'hot' red peppers, capsaicin [2]. We previously reported that WIN55141-2 ((R)-(+)-[2,3-dihydro-5-methyl-3-[4-morpholinylmethyl] pyrrolo [1,2,3-de]-1,4-benzoxazin-6-yl](1-naphthyl) methanone mesylate), a high-affinity cannabinoid receptor agonist, produced a dose-dependent inhibition of the number of capsaicin-induced coughs in mice [3]. This antitussive effect of WIN 55212-2 was antagonized by pretreatment with N-(piperidin-1-yl)-5-(4-chlorophenyl)-1-(2,4-dichlorophenyl)-4-methyl-1H-pyrazole-3-carboxamide hydrochloride (SR141716A), a cannabinoid CB1 receptor antagonist [3]. Therefore, we suggested that cannabinoid CB1 receptors play an important role in mediating the antitussive effect of cannabinoids [3]. Calignano et al [4] reported that inhaled anandamide inhibited capsaicin-induced cough and this effect was antagonized by pretreatment with SR141716A, a cannabinoid CB1 antagonist in guinea pigs. In contrast, Jia et al [5] reported that anandamide, when given by aerosol, induced coughs in conscious guinea pigs in a concentration-dependent manner. When guinea pigs were pretreated with capsazepine, the number of anandamide-induced coughs was significantly inhibited [5]. These results suggest that anandamide may inhibit and enhance capsaicin-induced cough through the activation of cannabinoid CB1 receptors and TRPV1 receptors respectively. However, the details of the mechanisms for the regulation of cough sensitivity by anandamide are not yet well known. De Petrocellis et al [6] demonstrated that anandamide increased the cytosolic Ca^2+ ^concentration in TRPV1-overexpressing HEK293 cells, and this effect was blocked by the TRPV1 antagonist capsazepine. Furthermore, N-arachidonoyl-(2-methyl-4-hydroxyphenyl) amine (VDM11), a selective anandamide membrane transporter inhibitor, strongly inhibited the effect of anandamide on the cytosolic Ca^2+ ^concentration in TRPV1-overexpressing HEK293 cells. Based on these results, they suggested that anandamide may play a role as an endovanilloid, which acts on the TRPV1 receptor at an intracellular site [6]. Thus, it is reasonable to consider that anandamide membrane transporters may play an important role in the modulation of coughsensitivity via the regulation of C-fiber activities.

To test this hypothesis, we investigated the effects of VDM11, an anandamide membrane transporter inhibitor, on capsaicin- and anandamide-induced cough in mice.

## Methods

### Animals

Male ICR mice (Tokyo Animal Laboratory Inc., Tokyo, Japan) weighing approximately 30 g were used. The animals had free access to food and water in an animal room, which was maintained at 24 ± 1° with a 12-h light-dark cycle. These studies were carried out in accordance with the Declaration of Helsinki and/or with the guide for the care and use of laboratory animals as adopted by the committee on the care and use of laboratory animals of Hoshi University, accredited by the Ministry of Education, Culture, Sports, Science and Technology, Japan.

### Antitussive assay

The cough reflex was induced as previously described [7]. Mice were exposed to a nebulized solution of capsaicin (45 μM) under conscious and identical conditions using a body plethysmograph. Capsaicin was dissolved to a concentration of 30 μg/ml in a 10% ethanol and 10% Tween 80 saline solution. The solution was diluted with saline. Anandamide was dissolved in 10% DMSO. The mice were exposed for 3 min to capsaicin or anandamide 60 min before the administration of antitussive drugs to determine the frequency of control coughs (Cc). The mice were also exposed for 3 min to capsaicin or anandamide 60 min after the administration of drugs. The number of coughs produced per 3-min period of exposure to capsaicin was counted. The number of coughs produced after administration of the drugs (Ct) was compared with the number of control coughs (Cc). The antitussive effect was expressed as the % inhibition of the number of control coughs ((Cc - Ct)/Cc × 100).

### Drugs

VDM11 and AM251 were purchased from TCRIS (Bristol, UK). Anandamide and capsazepine was purchased from Sigma-Aldrich Inc. (St. Lousi, MO, USA). SR141716A was generously supplied by Sanofi Synthelabo Recherche (Montpellier, France). VDM11 was dissolved in PBS/Tween 80/ethanol (18:1:1 v/v) [8]. All other drugs were dissolved in 10% DMSO. SR141716A was injected intraperitoneally 30 min after the administration of VDM11. SR141716A was also administered by inhalation via an aerosol for 4 min before the application of capsaicin. SR141716A was injected intracerebroventricularly 10 min before the application of capsaicin. Either capsazepine, a selective TRPV1 receptor antagonist, or AM251, a selective CB1 receptor antagonist, was also administered by aerosol inhalation for 4 min before the application of anandamide. VDM11 was also administered subcutaneously 60 min before the application of anandamide.

### Statistics

Data are expressed as means ± S.E. The statistical significance of differences was assessed by the Mann-Whitney U-test to evaluate the antitussive effect. A level of probability of 0.05 or less was considered significant.

## Results

### Antitussive effects of VDM11 on capsaicin-induced coughs

**Figure 1:**
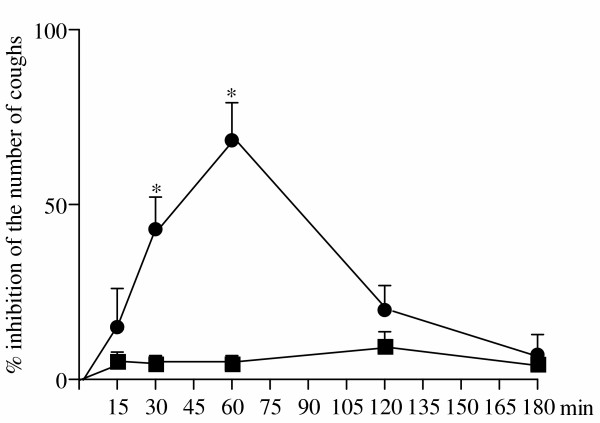
Time course of the antitussive effect of VDM11 (●) on capsaicin-induced coughs in mice. VDM11, at a dose of 10 mg/kg, was injected subcutaneously. Each point represents the mean with S.E. of 10 mice in each group. *P < 0.05 vs. respective vehicle (■ )-treated group.

**Figure 2:**
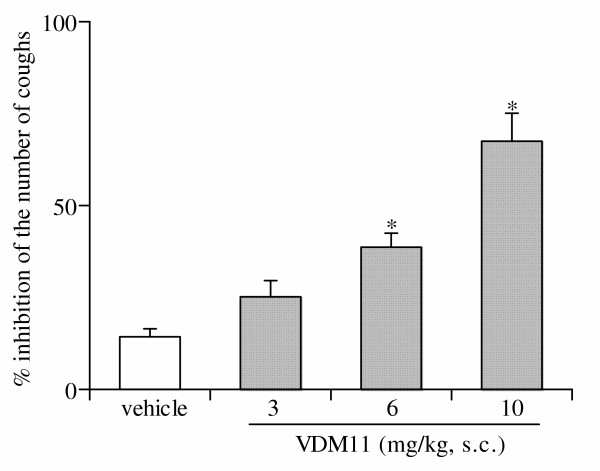
Dose-response relationship of the antitussive effect of VDM11 in mice. The antitussive effect of VDM11 was assessed 60 min after subcutaneous administration of VDM11. Each column represents the mean with S.E. of 10 mice in each group. *P < 0.05 vs. vehicle-treated group.

Exposure of control mice to a nebulized solution of capsaicin induced a reproducible cough response (17.3 ± 0.3 coughs/3 min; n = 101). Intraperitoneal injection of vehicle had no significant effect on the reproducibility of coughs over a period of 180 min after injection (Fig. 1). Antitussive effects of VDM11 (10 mg/kg subcutaneously) on capsaicin-induced cough reached a peak 60 min after injection, gradually declined and then returned to the pre-injection level 180 min after the administration of VDM11 (Fig. 1). Thus, a time interval of 60 min after the administration of VDM11 was chosen for experiments designed to quantify its effect. As shown in Fig. 2, VDM11 at doses of 3, 6 and 10 mg/kg, subcutaneously, produced a dose-dependent inhibition of the number of capsaicin-induced coughs (Fig. 2). Exposure to capsaicin (45 μM) 60 min before and after the administration of vehicle induced 16.6 ± 1.3 and 14.4 ± 1.2 coughs, respectively (Fig. 2). The effect of vehicle on the number of capsaicin-induced coughs was not significant.

### Effects of SR141716A on the antitussive effects of VDM11

**Figure 3:**
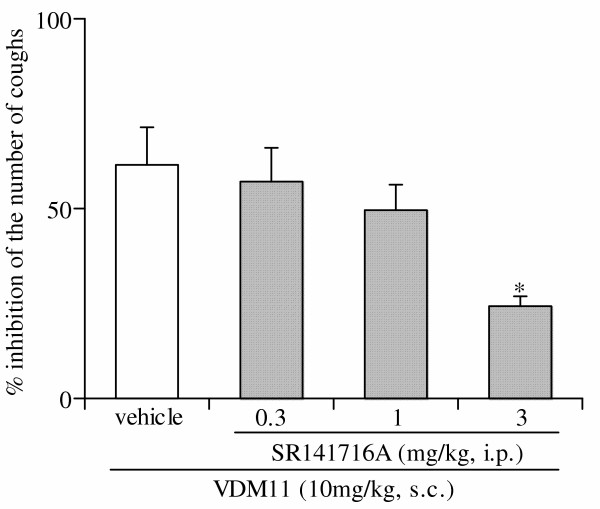
Effect of SR141716A on the antitussive effect of VDM11. SR141716A was injected intraperitoneally 30 min after the administration of VDM11. The antitussive effect of VDM11 (10 mg/kg) was assessed 60 min after administration. Each column represents the mean with S.E. of 7 mice in each group. *P < 0.05 vs. vehicle-treated group.

**Figure 4:**
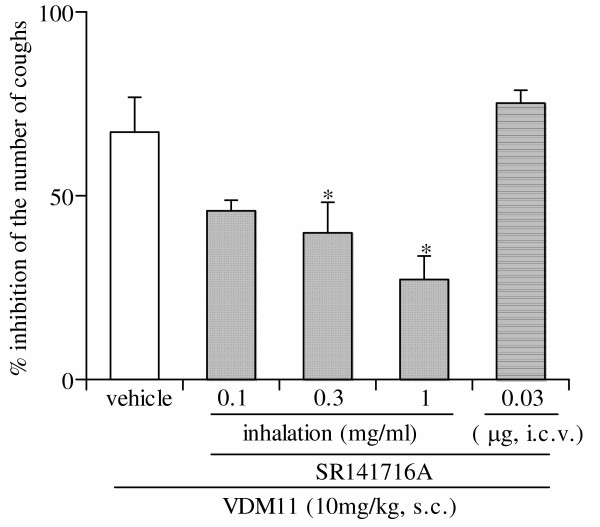
Effects of inhaled or intracerebroventricularly administered SR141716A on the antitussive effect of VDM11. SR141716A aerosol was inhaled for 4 min at 60 min after the administration of VDM11 (just prior to the second application of capsaicin). SR141716A was injected intracerebroventricularly, 50 min after VDM11 administration (10 min before the second application of capsaicin). The antitussive effect of VDM11 (10 mg/kg) was assessed 60 min after subcutaneous administration. Each column represents the mean with S.E. of 10 mice in each group. *P < 0.05 vs. vehicle-treated group.

Pretreatment with SR141716A, a cannabinoid CB1 receptor antagonist, at doses of 0.3, 1.0 and 3 mg/kg, intraperitoneally, dose-dependently antagonized the antitussive effect of VDM11 (10 mg/kg, subcutaneously) (Fig. 3). Furthermore, pretreatment with inhaled SR141716A at concentrations of 0.1, 0.3 and 1 mg/ml for 4 min also concentration-dependently suppressed the antitussive effect of VDM11 (10 mg/kg, subcutaneously) (Fig. 4). However, intracerebroventricular pretreatment with SR141716A, at a dose of 30 μg/mouse which was sufficient to block the CB1 receptor [9], had no effect on the antitussive effect of VDM11 (10 mg/kg, subcutaneously) (Fig. 4).

### Effect of anandamide on the cough reflex

**Figure 5:**
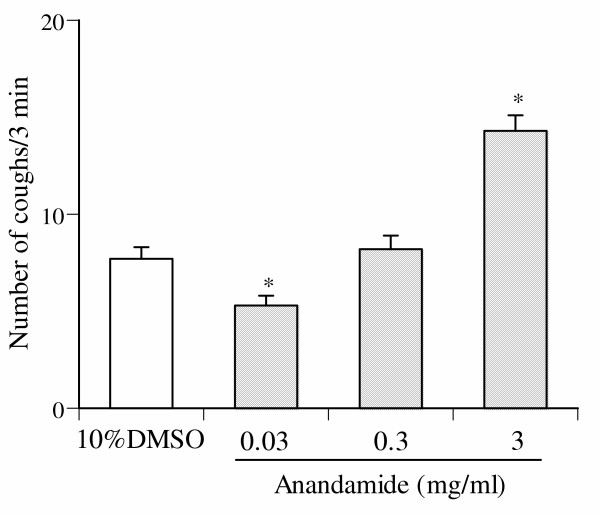
Effect of inhaled anandamide on the number of coughs. The number of coughs produced during 3-min exposure periods was counted. Each column represents the mean with S.E. of 10 mice in each group. *P < 0.05 vs. vehicle (10% DMSO)-inhaled group.

As shown in Fig. 5, exposure of mice to a nebulized solution of 10% DMSO, a vehicle of anandamide, induced a cough response (7.7 ± 0.6 coughs/3 min; n = 10). Exposure of mice to a nebulized solution of anandamide, at concentrations of 0.03, 0.3 and 3 mg/ml, also produced a cough response in a concentration-dependent manner (0.03 mg/ml, 5.3 ± 0.5 coughs/3 min, n = 10; 0.3 mg/ml, 8.1 ± 0.6 coughs/3 min; n = 10; 3 mg/ml, 14.3 ± 0.8 coughs/3 min; n = 10). The number of coughs induced by low dose (0.03 mg/ml) anandamide wassignificantly less than that of 10% DMSO. On the other hand, the number of coughs induced by high dose (3 mg/ml) anandamide was significantly greater than that of 10% DMSO.

### Effect of AM251 on low dose of anandamide-induced cough responses

**Figure 6:**
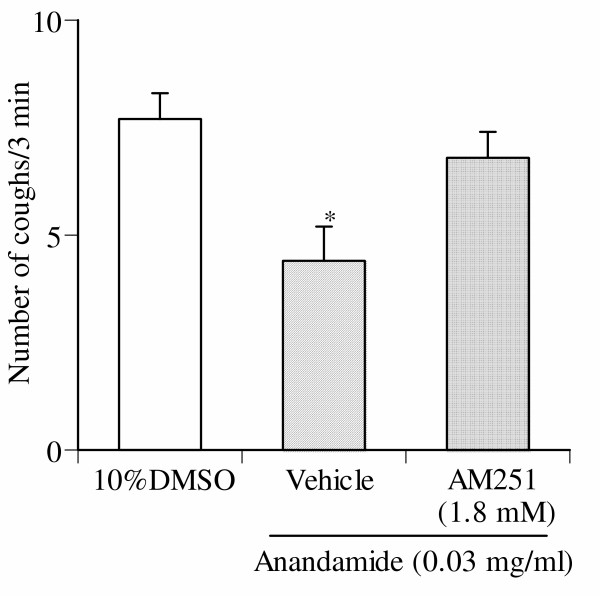
Effect of AM251 on low dose (0.03 mg/ml) anandamide-induced reduction of the number of coughs. AM251 was administered via aerosol for 4 min before the inhalation of anandamide. Each column represents the mean with S.E. of 10 mice in each group. *P < 0.05 vs. vehicle (10% DMSO)-inhaled group.

When AM251 (1.8 mM), a selective CB1 receptor antagonist, was given by aerosol for 4 min before inhalation of 0.03 mg/ml of anandamide, the number of coughs was significantly increased to the levels observed with 10% DMSO alone (Fig. 6).

### Effect of capsazepine and VDM11 on high dose of anandamide-induced cough responses

**Figure 7:**
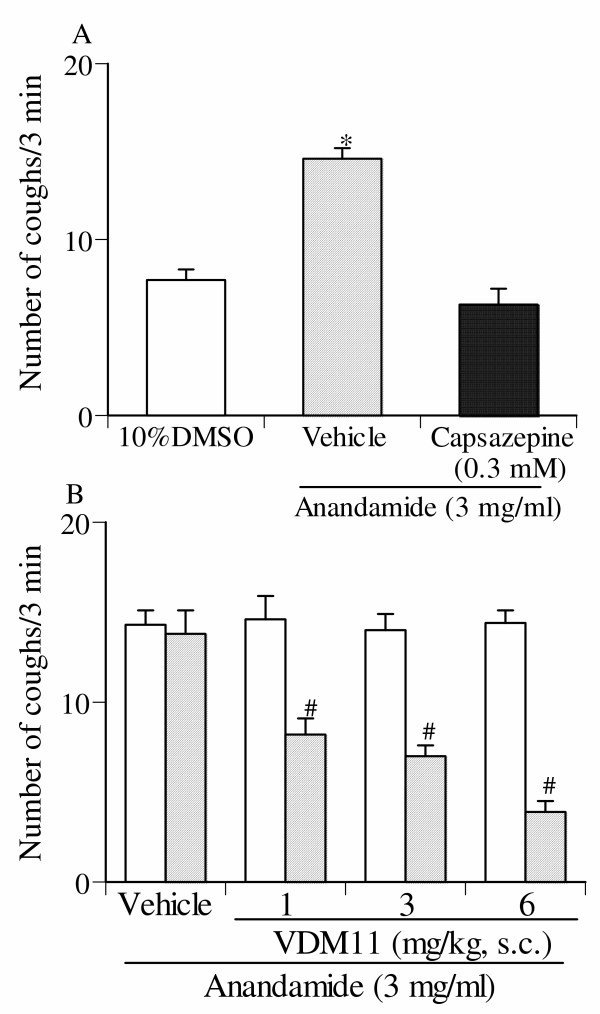
Effect of capsazepine (A) and VDM11 (B) on high dose (3 mg/ml) anandamide-induced reduction of the number of coughs. Capsazepine was given by aerosol for 4 min before the inhalation of anandamide. The antitussive effect of VDM11 (10 mg/kg) was assessed 60 min after subcutaneous administration. Each column represents the mean with S.E. of 10 mice in each group. *P < 0.05 vs. vehicle (10% DMSO)-inhaled group. #P < 0.05 vs. respective pre-value.

When capsazepine (0.3 mM), a selective TRPV1 receptor antagonist, was given via aerosol for 4 min before inhalation of 3 mg/ml of anandamide, the number of coughs was significantly decreased to the levels observed with 10% DMSO alone.(Fig. 7A). Furthermore, as shown in Fig. 7B, the number of coughs induced by high dose (3 mg/ml) anandamide was significantly and dose-dependently reduced by the pretreatment with VDM11. Indeed, when VDM11, at a dose of 3 mg/kg subcutaneously, was used as pretreatment, the number of coughs was significantly decreased to the level observed with 10% DMSO alone.

## Discussion

This study is the first to report that VDM11, a selective anandamide membrane transporter inhibitor, dose-dependently and significantly reduced the number of capsaicin-induced coughs. This antitussive effect of VDM11 was significantly antagonized by pretreatment with SR141716A, a cannabinoid CB1 receptor antagonist. These results suggest that the antitussive effect of VDM11 is mediated by the activation of cannabinoid CB1 receptors. We previously demonstrated that WIN 55212-2, a cannabinoid receptor agonist, had an antitussive effect through the activation of cannabinoid CB1 receptors [3]. Furthermore, we also suggested that the antitussive effect of WIN 55212-2 may depend mainly on central mechanisms [3]. In the present study, we observed that the antitussive effect of VDM11 was antagonized by systemic intraperitoneal or inhaled pretreatment with SR141716A. However, when SR141716A was injected intracerebroventricularly, the antitussive effect of VDM11 was not antagonized. These results suggest that VDM11 exerts its antitussive effect through an activation of peripheral CB1 receptors.

However, it is not reasonable to consider that VDM11 directly activates CB1 receptors. Giuffrida et al [10] reported that systemic administration of AM404, an anandamide membrane transporter inhibitor, caused a gradual increase in anandamide in rat plasma. Nemeth et al [11] reported that a low concentration of anandamide inhibited the release of substance P from peripheral capsaicin-sensitive sensory nerve terminals via the activation of cannabinoid CB1 receptors. Recently, we reported that NS-398, a selective cyclooxygenase-2 inhibitor, exerted peripheral antitussive effects by inhibiting the release of substance P from capsaicin-sensitive afferent C-fibers in airways [12]. Thus, it seems likely that inhibition of the release of substance P from capsaicin-sensitive sensory C-fibers may cause inhibition of the cough reflex. It has been reported that capsaicin binds to the cytosolic domain of TRPV1 receptors because of its lipophilic nature [13]. Substance P, which is contained in afferent C-fiber endings within the airway epithelium and smooth muscle layer, is released by activation of afferent C- fibers. Bonham et al [14] reported that substance P stimulates rapidly adapting receptors in guinea pigs. The stimulation of the rapidly adapting receptors by substance P is a potential link between the two airway defense systems, both of which elicit bronchoconstriction, mucus secretion and cough. Such a link, whereby C-fiber-receptor stimulation lead to the release of substance P and the subsequent stimulation of rapidly adapting receptors, has been previously proposed to explain the overlap of stimuli and reflex effects of both afferent systems [15]. Namely, that activation of TRPV1 receptors by capsaicin causes the release of substance P and the subsequent stimulation of rapidly adapting receptors, which may enhance cough reflexes. Based on these findings, it is possible that VDM11 accumulates endogenous anandamide, which results in the stimulation of cannabinoid CB1 receptors and the subsequent inhibition of substance P release from capsaicin-nensitive afferent C-fibers, which may produce the antitussive effect.

The above mentioned possibilities are further supported by the results of the present study. We observed that anandamide alone produced a biphasic modulation of the cough reflex. Indeed, the number of coughs induced by the inhalation of lower dose of anandamide (0.03 mg/ml) was significantly less than those induced by the inhalation of vehicle alone. On the other hand, the number of coughs induced by the inhalation of higher doses of anandamide (3 mg/ml) was significantly greater than those induced by the inhalation of vehicle alone. We also observed that when the animals were pretreated with AM251, a selective CB1 receptor antagonist, the number of coughs induced by the inhalation of lower dose of anandamide (0.03 mg/ml) was increased to the level of those induced by the inhalation of vehicle alone. This result suggests that activation of CB1 receptors by anandamide causes an inhibitory modulation of the cough reflex. Furthermore, it is possible that higher doses of anandamide may cause the activation of the cytosolic domain of TRPV1 receptors, since anandamide-induced enhancement of the number of coughs was abolished by pretreatment with either capsazepine, a selective TRPV1 receptor antagonist, or VDM11.

In conclusion, the present results suggest that anandamide, an endogenous cannabinoid ligand, may modulate cough sensitivity and anandamide transporters play an important role in this modulation. Furthermore, these findings indicate that inhibition of the uptake of anandamide produced a potent antitussive effect and suggest that the anandamide transporter is a potential target for peripherally acting antitussive drugs.
